# Stearoyl-CoA Desaturase-2 in Murine Development, Metabolism, and Disease

**DOI:** 10.3390/ijms21228619

**Published:** 2020-11-16

**Authors:** Lucas M. O’Neill, Chang-An Guo, Fang Ding, Yar Xin Phang, Zhaojin Liu, Sohel Shamsuzzaman, James M. Ntambi

**Affiliations:** 1 Department of Biochemistry, College of Agricultural and Life Sciences, University of Wisconsin-Madison, 433 Babcock Drive, Madison, WI 53706, USA; lmoneill@wisc.edu (L.M.O.); cguo36@wisc.edu (C.-A.G.); yphang@wisc.edu (Y.X.P.); zliu572@wisc.edu (Z.L.); mshamsuzzama@wisc.edu (S.S.); 2 Key Laboratory of RNA Biology, Institute of Biophysics, Chinese Academy of Sciences, Beijing 100101, China; dingfang0707@ibp.ac.cn; 3 University of Chinese Academy of Sciences, Beijing 100049, China; 4 Department of Nutritional Sciences, College of Agricultural and Life Sciences, University of Wisconsin-Madison, 1415 Linden Drive, Madison, WI 53706, USA

**Keywords:** Stearoyl-CoA Desaturase-2, preadipocyte differentiation, development, bone mineral density, vascular calcification, CNS diseases

## Abstract

Stearoyl-CoA Desaturase-2 (SCD2) is a member of the Stearoyl-CoA Desaturase (SCD) family of enzymes that catalyze the rate-limiting step in monounsaturated fatty acid (MUFA) synthesis. The MUFAs palmitoleoyl-CoA (16:1n7) and oleoyl-CoA (18:1n9) are the major products of SCD2. Palmitoleoyl-CoA and oleoyl-CoA have various roles, from being a source of energy to signaling molecules. Under normal feeding conditions, SCD2 is ubiquitously expressed and is the predominant SCD isoform in the brain. However, obesogenic diets highly induce SCD2 in adipose tissue, lung, and kidney. Here we provide a comprehensive review of SCD2 in mouse development, metabolism, and various diseases, such as obesity, chronic kidney disease, Alzheimer′s disease, multiple sclerosis, and Parkinson′s disease. In addition, we show that bone mineral density is decreased in SCD2KO mice under high-fat feeding conditions and that SCD2 is not required for preadipocyte differentiation or the expression of PPARγ in vivo despite being required in vitro.

## 1. Introduction

Stearoyl-CoA Desaturase-2 (SCD2) is a member of the Stearoyl-CoA Desaturase (SCD) family of enzymes that catalyze the de novo synthesis of monounsaturated fatty acids (MUFAs). The SCD enzymes introduce a single double bond at the Δ9,10 position of long-chain fatty acyl-CoAs that are synthesized de novo or obtained from the diet [1]. There are four SCD isoforms in mouse (SCD1–4) and two in humans (hSCD1&5) [2]. Of the murine SCD isoforms, SCD1 and SCD2 are the most similar in sequence, substrate specificity, and tissue expression [3]. All SCD isoforms are integral enzymes that reside in the endoplasmic reticulum (ER) [4]. The topology of the SCD isoforms consists of four transmembrane domains and three loops connecting them [4]. Both the NH_2_ and COOH termini are oriented toward the cytosol. To desaturate SFAs, SCD requires NAD(P)H as an electron donor and oxygen as a final electron acceptor. Within the SCD enzyme, there are three conserved histidine motifs that bind Fe^2+^, which in turn activates molecular oxygen. During the process of desaturation, electrons flow from NAD(P)H to cytochrome b_5_ reductase (FADH2), to cytochrome b_5_, to SCD, and finally to O_2_; which is reduced to H_2_O [1,5,6].

SCD2 converts mainly palmitoyl-CoA (16:0) and stearoyl-CoA (18:0) to palmitoleoyl-CoA (16:1n7) and oleoyl-CoA (18:1n9), respectively [3]. Palmitoleoyl-CoA and oleoyl-CoA are a source of energy and are major components of membrane phospholipids (PL), triglycerides (TG), cholesterol esters (CE), and wax esters [7]. Furthermore, palmitoleoyl-CoA and oleoyl-CoA have been shown to act as signaling molecules. Adipose-derived palmitoleoyl-CoA (16:1n7) suppresses hepatic lipogenesis and increases hepatic and muscle insulin sensitivity [8]. Hepatic oleoyl-CoA (18:1n9) directly associates with and activates the lipogenic transcription factor sterol regulatory element-binding protein-1c (SREBP1c), which, in turn, activates fatty acid synthase (FAS), carbohydrate response element-binding protein (ChREPB), and SCD itself [9]. Furthermore, oleoyl-CoA attenuates saturated fatty acid-induced ER stress by suppressing the mammalian target of rapamycin (mTOR) via peroxisome proliferator-activated receptor gamma coactivator-1 alpha (PGC-1a) [10]. Lastly, oleoyl-CoA, administered directly to the hypothalamus, reduces appetite and hepatic gluconeogenesis [11].

SCD2 was first identified in 1989 by James Ntambi and was characterized by Klaus Kaestner in M. Daniel Lane′s laboratory at The Johns Hopkins University [12]. Ntambi was using a variety of probes to detect the stearoyl-CoA desaturase-1 (*Scd1*) gene in differentiated 3T3-L1 preadipocytes. However, one probe detected an unexpected band that he identified to be an isoform of *Scd1* and named *Scd2* [12]. Soon after, Kaestner and Ntambi determined that *Scd2* was highly expressed in the brain and induced in adipose tissue, kidney, and lung when mice were fed a lipogenic high-carbohydrate diet (HCD) [12]. Today, we know that a high-fat diet (HFD) also induces *Scd2* in adipose tissue and that *Scd2* is expressed ubiquitously at detectable levels except in the adult liver [13,14]. Furthermore, in addition to the brain, *Scd2* is the predominant isoform in B lymphocytes, epidermis, lung, pancreas, spleen, testis, and ovaries [15,16,17,18].

Since the first published report on SCD2 31 years ago, researchers have made great progress in determining the physiological roles of SCD2. To do this, multiple gene targeting strategies have been utilized, from siRNA to CRE/LOX, to inactivate *Scd2;* as well as lentiviral vectors to overexpress *Scd2*. Here, we discuss the role of SCD2 in murine development, adiposity, energy homeostasis, various diseases, and human relevance. We also provide new evidence that shows (1) *Scd2* deficient mice fed a high-fat diet have decreased bone mineral density and that (2) SCD2 is not required for preadipocyte differentiation or the expression of PPARγ in vivo despite being required in vitro.

## 2. Results and Discussion

### 2.1. The Role of SCD2 in Developing Mice

**Figure 1:**
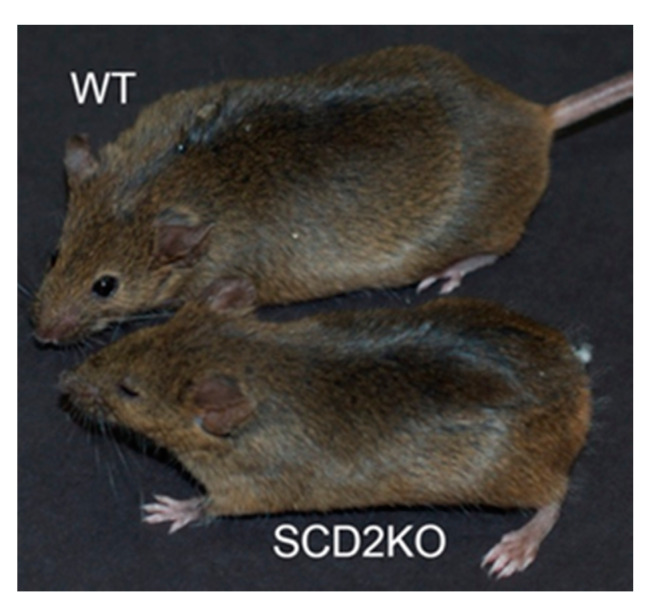
Visual comparison of wild-type (WT) (top) and Stearoyl-CoA Desaturase-2 deficient (SCD2KO) (bottom) mice in the 129/Sv genetic background.

SCD2 is required for the survival, development, and de novo synthesis of MUFAs in young mice [14]. Using the Cre/Lox gene inactivation strategy, our laboratory created *Scd2* deficient mice (SCD2KO) in both the C57BL/6 and 129/SV genetic backgrounds [14]. In the C57BL/6 genetic background, *Scd2* deficiency was 100% embryonic lethal. In the 129/SV genetic background, >70% of SCD2KO mice died within 24 h of birth [14]. Moreover, newborn SCD2KO mice were smaller than wild-type (WT) mice, had kinky tails, and their skin appeared dry and cracked [14]. The SCD2KO mice that survived to adulthood were fertile, retained their kinky tail phenotype, and remained smaller than WT mice (Figure 1). It is worth noting that *Scd2^+^/^−^* mice are haplosufficient and resemble WT mice phenotypically [14].

In the 129/SV genetic background, deletion of *Scd2* creates a defective skin barrier which results in trans-epidermal water loss and ultimately leads to the death of newborn mice [14]. Skin structure analysis shows a decreased number of lamellar bodies forming in the epidermis layer of the SCD2KO mice. Lamellar bodies are secreted by the keratinocytes, the most abundant cell type in the epidermis, to produce a lipid-containing membrane that serves as a water barrier [19]. *Scd2* deficiency decreases the contents of lamellar bodies and changes the lipid composition of the epidermis [14]. The epidermis of SCD2KO mice displays significantly decreased MUFAs in the triglyceride (TG), cholesterol ester (CE), acylceramide (AC), phospholipid (PL), and free fatty acid (FFA) fractions. Linoleic acid (18:2n6) ACs are the major lipid species involved in protecting the epidermis from permeability [20,21]. Although the total epidermal linoleic acid content is similar between SCD2KO and WT mice, *Scd2* deficiency decreases the amount of linoleic acid (18:2n6) in the epidermal AC fraction, which is consistent with a defective skin barrier [20,21]. In SCD2KO mice, we hypothesized that linoleic acid from ACs is repartitioned to PLs to compensate for decreased MUFAs and to maintain proper membrane fluidity and cell structure.

Besides altering epidermal lipid composition, SCD2 deficiency may inhibit the differentiation of keratinocytes. In the epidermis of one day old SCD2KO mice, the markers for keratinocyte differentiation, transglutaminase-1 and involucrin, are significantly reduced [14]. Other skin barrier associated genes were unchanged, such as β-cerebrosidase (*Gba*) and acyl-CoA:diacylglycerol acyltransferase-2 (*Dgat2*). Compared to *Scd2*, *Scd1* is predominantly expressed in the dermis of WT mice; however, *Scd1* expression is induced in the epidermis and dermis of newborn SCD2KO mice, likely to compensate for the loss of *Scd2.* On the other hand, expression of *Scd3* was induced in the epidermis but not dermis of SCD2KO mice. Again, we hypothesized that this occurs to compensate for the loss of *Scd2*. Both *Gba* deficient (GBAKO) and *Dgat2* deficient mice (DGAT2KO) exhibit skin barrier defects and high neonatal lethality similar to the SCD2KO mice [22,23]. Moreover, they highlight the importance of early lipid synthesis in development and survival. Unlike the SCD2KO mice that only survive in the 129 genetic background, DGAT2KO and GBAKO mice can survive in a mixture of 129/SV and C57BL/6 genetic backgrounds [22,23]. It is currently unknown why a genetic background affects these knockout mice differently.

In mice, from embryos until 21 days old, *Scd2* is highly expressed in the liver, and *Scd1* expression is absent [14]. In contrast, *Scd2* is absent in the adult liver, and *Scd1* is highly expressed. Therefore, SCD2 is a critical enzyme that controls triglyceride synthesis in young mice. In SCD2KO mice less than 21 days old, hepatic SCD activity was reduced by 70% leading to a decrease in MUFAs, including 16:1n7, 18:1n9, and vaccenic acid (18:1n7). Moreover, the levels of hepatic and plasma TGs, as well as hepatic TG synthesis were significantly decreased in the newborn SCD2KO mice. Interestingly, *Scd1* and *Scd2* expression switched when the mice were 21 days old. Given that weaning occurs during this time, maternal milk may induce *Scd1* and repress *Scd2* transcription, but this has yet to be investigated.

### 2.2. SCD2 and Bone Density

**Figure 2:**
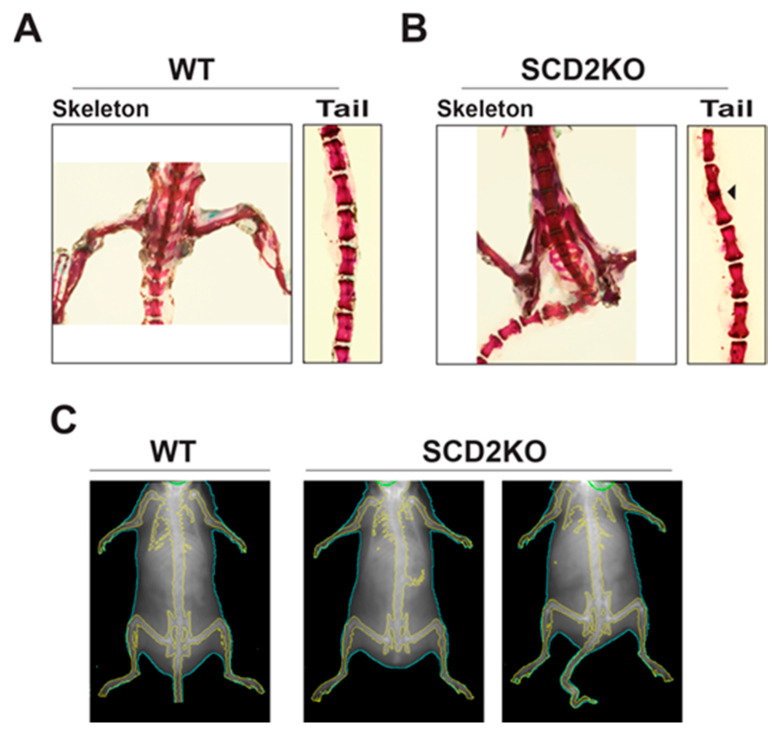
(**A**) Skeleton and caudal vertebrae of a WT mouse stained with alizarin red. (**B**) Skeleton and caudal vertebrae of an SCD2KO mouse stained with alizarin red. (**C**) Dual-energy X-ray absorptiometry (DEXA) images comparing WT (left) and SCD2KO mice (middle and right).

**Figure 3:**
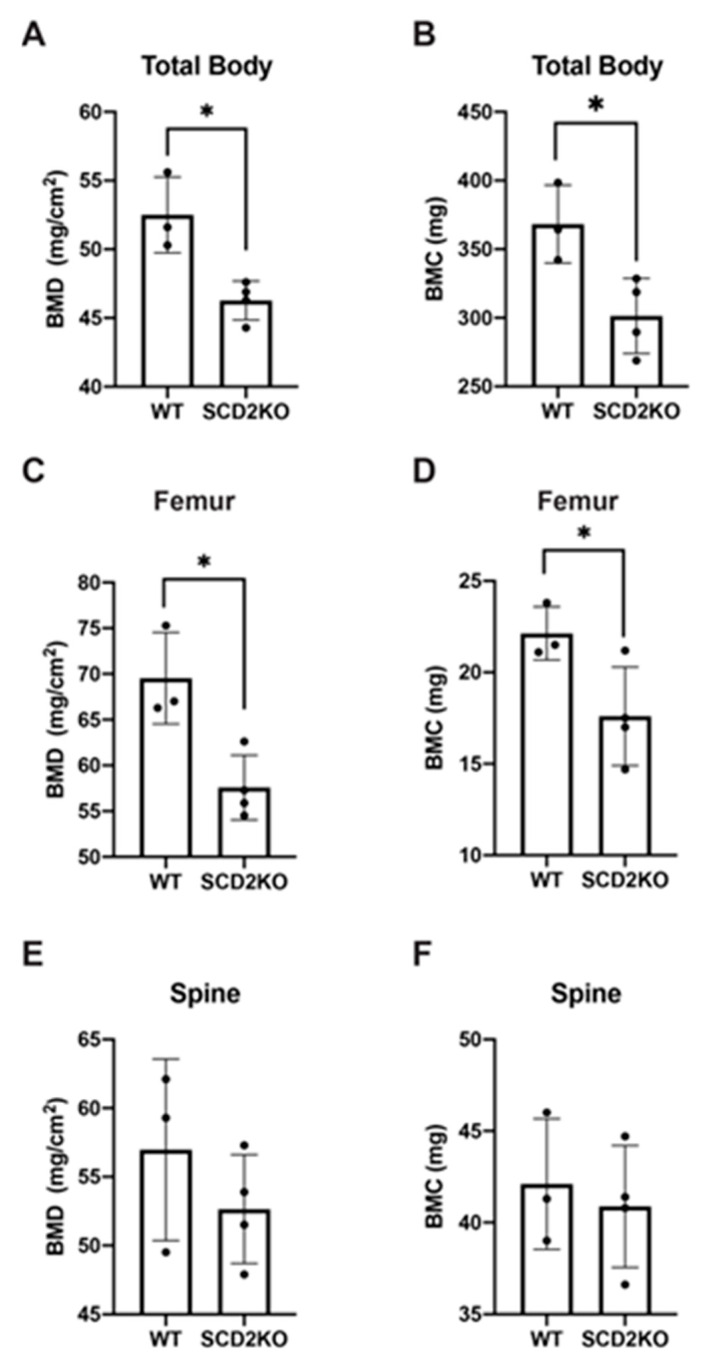
SCD2KO mice have decreased bone mineral density (BMD) and bone mineral content (BMC). (**A**) Total BMD of male WT (*n* = 3) and SCD2KO (*n* = 4) mice, fed 10 weeks high-fat diet (HFD). (**B**) Total BMC of male WT (*n* = 3) and SCD2KO (*n* = 4) mice, fed 10 weeks HFD. (**C**) Femoral BMD of male WT (*n* = 3) and SCD2KO (*n* = 4) mice, fed 10 weeks HFD. (**D**) Femoral BMC of male WT (*n* = 3) and SCD2KO (*n* = 4) mice, fed 10 weeks HFD. (**E**) Spinal BMD of male WT (*n =* 3) and SCD2KO (*n* = 4) mice, fed 10 weeks HFD. (**F**) Spinal BMC of male WT (*n* = 3) and SCD2KO (*n* = 4) mice, fed 10 weeks HFD. Values are mean ± SD, * *p* < 0.05 vs. WT by Student′s two-tailed *t*-test. Dots represent individual values.

SCD2KO mice have a skeletal phenotype that is most prominently observed in the tail. The tails of SCD2KO mice can have a single caudal vertebrae fusion, multiple fusions, or be completely absent (Figure 2A–C). The link between *Scd2* deficiency and bone development is currently unknown and warrants further research. Given that SCD2KO mice have defects in bone development and remain lean when consuming a HFD, we hypothesized that SCD2KO mice have altered bone mineral density [14,24]. Obesity has been shown to have a protective effect on bones because increased mass on the skeleton leads to increased bone mineral density (BMD) [25,26]. To test if BMD is altered in SCD2KO mice, we assessed total, femoral, and spinal bone mineral density (BMD) and bone mineral content (BMC) by dual-energy X-ray absorptiometry (DEXA). Our results showed that SCD2KO mice fed HFD had significantly decreased total and femoral BMD and BMC compared to WT controls (Figure 3A–D). Surprisingly, spinal BMD and BMC were unchanged (Figure 3E,F). Overall, the decreased BMD exhibited by the SCD2KO mice is consistent with them being leaner than WT mice. However, we cannot rule out that bone development or physical effects may also contribute to decreased BMD. The tail bone deformities in the SCD2KO mice may inhibit their mobility, which could lead to decreased femoral BMD. Furthermore, because SCD2KOs are smaller than WT mice, it is expected that femoral and total BMC will be decreased. Spinal BMD and BMC in the SCD2KO mice may be similar because caudal vertebrae were excluded from the scan and also their thoracic vertebrae are similar in size to WT controls. Future research is needed to evaluate if SCD2 directly affects in bone mineral density during bone development and the role of SCD2 in bone health.

### 2.3. The Relationship between SCD2, Adiposity, and Energy Expenditure

Recently, we showed that SCD2 is an important part of whole-body energy homeostasis in adult mice. We reported that SCD2KO mice are protected from both HFD and HCD-induced adiposity [24]. Likewise, de Moura and colleagues showed that the targeted knockdown of *Scd2* in the hypothalamus of obese mice blunts weight gain [27].

When SCD2KO mice are fed either HFD or HCD, they fail to gain weight compared to WT mice [24]. At the end of these feeding studies, relative fat mass was decreased in the SCD2KO mice. In addition, HFD-fed SCD2KO mice have increased glucose and insulin tolerance corresponding to their lean phenotype. Protection against diet-induced adiposity in the SCD2KO mice was not due to decreased food consumption or dietary lipid absorption as both groups of mice ate similar levels of food and had similar levels of fecal FFAs. The markers for thermogenesis, peroxisome proliferator-activated receptor gamma, coactivator 1 alpha (*Pgc1-*α), and uncoupling protein-1 (*Ucp1)* were increased in the brown adipose tissue (BAT) of SCD2KO mice compared to WT mice. However, when energy expenditure was measured by indirect calorimetry, there was no difference between SCD2KO mice and WT mice [24].

In support of SCD2 altering energy homeostasis, knockdown of *Scd2* in the hypothalamus of obese mice results in increased energy expenditure [27]. In this study, Swiss mice were made obese by feeding them HFD for six weeks. Once obese, *Scd2* was knocked down in the hypothalamus by intraperitoneal injection of antisense oligonucleotides (SCD2ASO) or by direct hypothalamic injection of lentiviral shSCD2 (shSCD2). SCD2ASO treatment blunted weight gain and increased relative oxygen consumption and spontaneous locomotion. Conversely, the average change in body weight of obese mice treated with shRNA was not statistically different from control mice. Overexpression of hypothalamic *Scd2* did not have any effect on body mass. When comparing the SCD2KO, SCD2ASO, and shSCD2 mice, it is likely that multiple tissues, such as white adipose tissue (WAT), BAT, and the hypothalamus are all contributing to the phenotypes created by *Scd2* deficiency.

Lastly, the protection against diet-induced adiposity and altered thermogenesis caused by *Scd2* deficiency and hypothalamic knockdown of *Scd2* is mild when compared with SCD1KO mice or mice with a skin-specific deletion of *Scd1* (SKO) [28,29,30]. SCD1KO and SKO mice are hypermetabolic and severely cold intolerant due to skin barrier abnormalities that promote heat and water loss [31]. SCD1KO and SKO mice also have dry skin, alopecia, and sebocyte hypoplasia [30]. These skin barrier defects do not occur in adult SCD2KO mice; therefore, a different mechanism must drive the beneficial metabolic effects of SCD2 deficiency.

### 2.4. The Role of SCD2 in Preadipocyte Differentiation In Vitro and In Vivo

In mice, *Scd1* is the most highly expressed SCD isoform in adipose tissue; however, obesogenic diets, such as HFD or HCD, highly induce *Scd2* in adipose tissue [12,13,32]. In mice fed HFD, *Scd2* mRNA levels are significantly higher than 26 other enzymes involved in lipid metabolism [15]. One hypothesis that explains this is that increased SCD2 expression promotes preadipocyte differentiation and increases the number of adipocytes available for FA storage during times of excess energy consumption. Another hypothesis is that SCD2 induction increases the storage of SFA in the form of TGs. Supporting the latter hypothesis, reports show SCD1 co-localizes with DGAT2, an ER enzyme responsible for synthesizing the final step in TG synthesis in which a fatty acyl-CoA is added to diacylglycerol to form TG. [33]. Therefore, due to the proximity between SCD1 and DGAT2, DGAT2 prefers to synthesize TGs using fatty acyl-CoAs synthesized by SCD1. Given the high degree of similarity between SCD1 and SCD2, it is very likely SCD2 and DGAT2 work together similarly to SCD1 and DGAT2.

In support of the former hypothesis, data suggests that SCD2 is required to differentiate 3T3-L1 preadipocytes into mature adipocytes in vitro [15]. siRNA mediated knockdown of SCD2 inhibited the transcription of peroxisome proliferator-activated receptor gamma (*Pparg*), reduced PPARγ protein levels by 90%, and suppressed adipocyte lipid accumulation. PPARγ is a nuclear lipogenic transcription factor and master regulator of adipogenesis [34]. PPARγ is responsible for inducing the expression of multiple downstream genes, such as CCAAT/Enhancer binding protein (*Cebp*), fatty acid synthase (*Fasn*), acetyl-CoA carboxylase (*Acc*), *Scd1*, and *Dgat2* [35,36,37]. The addition of rosiglitazone, a PPARγ-specific ligand, does not rescue differentiation in preadipocytes treated with SCD2 siRNA, suggesting that SCD2 is not responsible for synthesizing a PPARγ-specific ligand. Interestingly, adding the PPARγ agonist troglitazone does not alter *Scd2* expression levels in preadipocytes, but the addition suppresses *Scd1* expression in mature adipocytes [38]. This suggests that PPARγ ligands may affect SCD expression in different ways. Furthermore, although PPARγ induction requires SCD2 during differentiation, PPARγ may not be a potent regulator of SCD2, despite SCD2 having a PPAR response element-binding site. In mature post-differentiated adipocytes, knockdown of SCD2 only inhibits PPARγ translation but not transcription or degradation [15]. As PPARγ translation decreases, the expression of PPARγ target genes also decreases. Therefore, SCD2 is required to maintain the phenotype of mature adipocytes in vitro.

Despite the requirement for SCD2 in 3T3-L1 differentiation, SCD2KO mice are not lipodystrophic and have developed fat pads [24]. This suggests that although *Scd2* deficiency could affect adipogenesis, SCD2 is not required for preadipocyte differentiation and PPARγ expression in vivo. To address this discrepancy, we assessed preadipocyte differentiation and PPARγ levels in differentiated SCD2KO primary preadipocytes, *Scd2* deficient 3T3-L1 cells, and adipose depots from SCD2KO mice. First, we confirmed that the off-target effects of SCD2 siRNA did not contribute to the inhibition of preadipocyte differentiation. To do this, we knocked out *Scd1* and *Scd2* in 3T3-L1 cells using CRISPR and induced preadipocyte differentiation using a cocktail of insulin, dexamethasone, and 3-Isobutyl-1-methylxanthine. Similar to the work conducted by Christianson et al., we report that *Scd*2 inhibits preadipocyte differentiation in vitro and it cannot be rescued by the addition of PPARγ agonists rosiglitazone and indomethacin.

**Figure 4:**
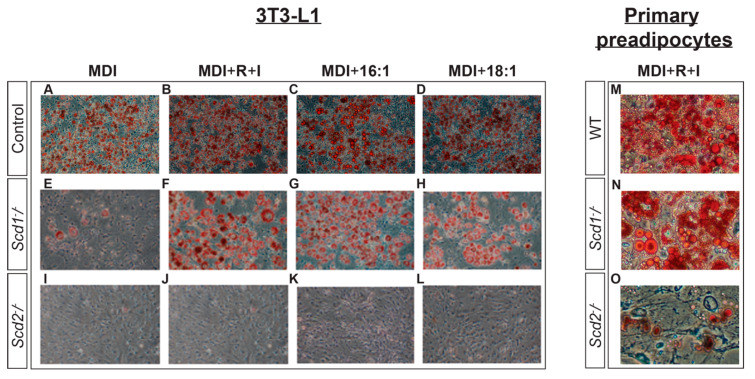
3T3-L1 cells and primary preadipocytes were stained with Oil Red O to visualize lipid accumulation 10 days after initiating preadipocyte differentiation, using methylisobutylxanthine, dexamethasone, and insulin (MDI), as described in the “materials and methods” section. (**A**) Control 3T3-L1 cells treated with MDI. (**B**) Control 3T3-L1 cells treated with MDI, rosiglitazone (R), and indomethacin (I). (**C**) Control 3T3-L1 cells treated with MDI and palmitoleic acid (16:1n7). (**D**) Control 3T3-L1 cells treated with MDI and oleic acid (18:1n9). (**E**) *Scd1^−^/^−^* 3T3-L1 cells treated with MDI. (**F**) *Scd1^−^/^−^* 3T3-L1 cells treated with MDI, rosiglitazone (R), and indomethacin (I). (**G**) *Scd1^−^/^−^* 3T3-L1 cells treated with MDI and palmitoleic acid (16:1n7). (**H**) *Scd1^−^/^−^* 3T3-L1 cells treated with MDI and oleic acid (18:1n9). (**I**) *Scd2^−^/^−^* 3T3-L1 cells treated with MDI. (**J**) *Scd2^−^/^−^* 3T3-L1 cells treated with MDI, rosiglitazone (R), and indomethacin (I). (**K**) *Scd2^−^/^−^* 3T3-L1 cells treated with MDI and palmitoleic acid (16:1n7). (**L**) *Scd2^−^/^−^* 3T3-L1 cells treated with MDI and oleic acid (18:1n9). (**M**) WT primary preadipocytes treated with MDI, rosiglitazone (R), and indomethacin (I). (**N**) SCD1KO primary preadipocytes treated with MDI, rosiglitazone (R), and indomethacin (I). (**O**) SCD2KO primary preadipocytes treated with MDI, rosiglitazone (R), and indomethacin (I).

After ten days of preadipocyte differentiation, both the *Scd2* and *Scd1* deficient cells did not accumulate lipid in the form of lipid droplets, compared to control cells, indicating that preadipocyte differentiation is impeded (Figure 4A,E,I). This is in contrast to siRNA mediated knockdown of *Scd1* and *Scd2*, where knockdown of *Scd2* but not *Scd1* inhibited preadipocyte differentiation and indicates that low expression levels of SCD1 may be sufficient to promote preadipocyte differentiation; however, this has yet to be investigated directly. In *Scd2* deficient cells, adding the PPARγ agonists rosiglitazone and indomethacin or SCD products oleic acid or palmitoleic acid did not restore preadipocyte differentiation and lipid droplet formation (Figure 4I–L). However, the addition of rosiglitazone and indomethacin, oleic acid, and palmitoleic acid, enhanced preadipocyte differentiation and lipid accumulation in the *Scd1* deficient cells, respectively (Figure 4E–H). As expected, control cells treated with rosiglitazone and indomethacin, oleic acid, and palmitoleic acid maintained high levels of differentiation and lipid accumulation (Figure 4A–D).

**Figure 5:**
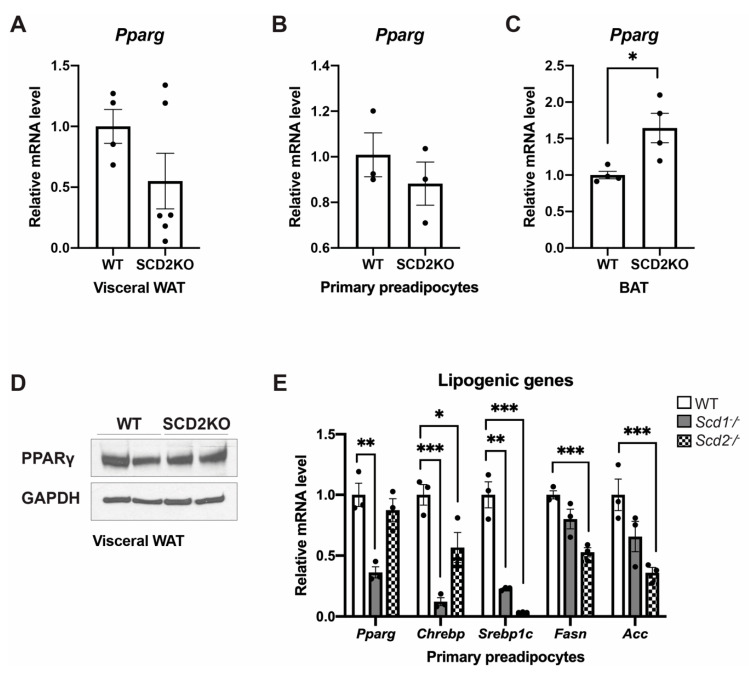
(**A**) Relative *Pparg* transcript levels in the visceral white adipose tissue (WAT) of WT (*n* = 4) and SCD2KO (*n* = 6) mice fed HFD for 10 weeks. (**B**) Relative *Pparg* transcript levels in differentiated primary preadipocytes from WT (*n* = 3) and SCD2KO (*n* = 3) mice. (**C**) Relative *Pparg* transcript levels in the brown adipose tissue (BAT) of WT (*n* = 3) and SCD2KO (*n* = 4) mice fed HFD for 10 weeks. (**D**) Immunoblot analysis of PPARγ in visceral WAT of WT (*n* = 2) and SCD2KO (*n* = 2) mice fed HFD for 10 weeks. (**E**) Relative lipogenic gene expression in WT (*n* = 3), SCD1KO (*n* = 3), and SCD2KO (*n* = 3) primary preadipocytes treated with MDI, rosiglitazone (R), and indomethacin (I). Values are mean ± SEM, * *p* < 0.05 vs. WT, ** *p* < 0.01 vs. WT, *** *p* < 0.005 vs. WT by Student′s two-tailed *t*-test. Dots represent individual values.

Primary preadipocytes from SCD2KO mice failed to differentiate and form lipid droplets compared to those from SCD1KO and WT mice (Figure 4M–O). More lipid droplets formed in differentiated SCD1KO and SCD2KO primary preadipocytes than *Scd1-*deficient and *Scd2*-deficient 3T3-L1 cells suggesting that other factors are contributing to preadipocyte differentiation in vivo that are absent in vitro. In both SCD1KO and SCD2KO primary preadipocytes, lipogenic gene transcript levels are decreased suggesting that although *Scd2* is required for primary preadipocyte differentiation, both *Scd1* and *Scd2* are needed for maintaining lipogenic gene expression (Figure 5E).

Both differentiated SCD2KO primary preadipocytes and visceral adipose tissue fat pads of SCD2KO mice have lower *Pparg* transcript levels, although they are not significantly different from WT controls (Figure 5A,B). Similarly, there is no change in PPARγ protein levels in the visceral adipose tissue fat pads of SCD2KO and control mice (Figure 5D). In the BAT of SCD2KO mice, *Pparg* transcript levels are significantly elevated (Figure 5C). Taken together, this suggests that SCD2 is not by itself required for PPARγ expression in vivo and is likely the reason preadipocyte differentiation is not ablated in SCD2KO mice.

### 2.5. SCD2 and Vascular Calcification

Vascular calcification (VC) is the leading cause of death in patients with chronic kidney disease (CKD) [39,40]. It is also prevalent in elderly patients and those with diabetes [41]. VC is caused by the differentiation of vascular smooth muscle cells (VSMCs) into osteoblast-like cells [42].

In murine models of VC, both SCD1 and SCD2 levels are significantly reduced [43]. Moreover, fully saturated phosphatidic acids (PAs) such as 1,2-distearoyl-PA (18:0/18:0-PA) have been shown to promote osteoblastic differentiation and mineralization of VSMCs by inducing activating transcription factor 4 (ATF4), a key transcription factor that mediates both osteoblastic differentiation and ER stress [44]. Increased synthesis of stearoyl-CoA by either supplementation of exogenous stearic acid or the addition of SCD inhibitor increases 1,2-distearoyl-PA (18:0/18:0-PA), which increases ER stress, induces ATF4, and promotes calcification [43,44]. In contrast, the presence of oleoyl-CoA reduces 1,2-distearoyl-PA (18:0/18:0-PA) levels and ultimately protects against calcification [43,44].

VSMCs express both SCD1 and SCD2 isoforms, and the simultaneous deletion of both isoforms are required for VC to occur [43]. Mice harboring a dual deletion of *Scd1* and *Scd2* in smooth muscle cells displayed severe VC in the aortic sinus and arch lesions compared to control mice [43]. Taken together, the onset of VC in mice is mediated by both SCD1&2 and maintaining the normal function of SCD1&2 is vital for protecting the kidney from chronic kidney disease. Therefore, kidney-specific SCD agonists may be a potential therapeutic for the treatment of VC in CKD.

### 2.6. SCD2 and Central Nervous System Diseases

It is well documented that SCD2 is highly expressed in the brain [12]. However, the role that SCD2 plays in brain function has not yet been thoroughly investigated in part because newborn SCD2KO mice have high levels of lethality. Despite the lack of research investigating SCD2 directly, there are indications that SCD2 may be a key player involved with various diseases of the central nervous system (CNS), such as Alzheimer′s disease, multiple sclerosis, and Parkinson′s Disease [45,46,47]. However, additional studies that address the impact of SCD2 in different brain regions are needed to understand whether it may serve as a new target against toxicity in human neurological disorders.

In humans, Alzheimer′s disease (AD) affects more than 35 million people worldwide [48]. AD is characterized by progressive memory impairment, deterioration of language, and visuospatial deficits [48]. In the brains of subjects pathologically confirmed to have AD, hSCD1 and hSCD5 expression were markedly elevated compared to control subjects [49]. Additionally, nervonoyl-CoA (C24:1n9) and other MUFA levels were also elevated [49]. Despite the correlation between AD and elevated SCD expression levels, there has been very little published research in this area. In 2007, a patent application by Myriad Genetics Inc. (US20070087363A1) showed that overexpression of hSCD1 in neuronal H4 cells leads to increased secretion of amyloid-beta42, an AD plaque-forming unit, and that inhibition of SCD significantly decreased amyloid-beta42 secretion [50]. Although it has not yet been tested, it is likely overexpression and inhibition of hSCD5 or SCD2 would have similar results and may lead to new treatments of AD. More research is needed to fully understand the role of SCDs in AD.

Multiple sclerosis (MS) is the most common cause of non-traumatic neurological disability in young adults [51,52]. MS is a chronic inflammatory and neurodegenerative disease affecting the central nervous system. In MS, myelin and the myelin-producing cells, oligodendrocytes, are destroyed [53]. Myelin is a lipid bilayer that protects axons from external damage and enhances neural signaling and transmission. SCD2 is the predominant SCD isoform expressed in oligodendrocytes [54]. After cholesterol, sphingomyelin is the next most abundant lipid species that comprise the myelin sheath [55]. The MUFA, nervonoyl-CoA, a downstream product of oleoyl-CoA, is the major FA component of sphingomyelin. To produce nervonoyl-CoA, the SCD2 product oleoyl-CoA is subsequently elongated by elongase-1 three times [56]. Thus, in mice, SCD2 predominately carries out the rate-limiting step of nervonoyl-CoA (C24:1n9) biosynthesis in oligodendrocytes [57].

In MS patients and experimental autoimmune encephalomyelitis rodents used as a model for MS, both sphingomyelin and nervonoyl-CoA are significantly decreased compared to control samples [57,58]. These data suggest that SCD2 is a critically important enzyme that controls the supply of nervonoyl-CoA (C24:1n9) for myelin biosynthesis and that inhibition of SCD2 may significantly contribute to MS [46]. In contrast, SCD1 inhibition or phagocyte-specific deficiency of *Scd1* accelerated remyelination ex vivo and in vivo [59]. Future research is needed to determine the cell-specific roles of SCD1 and SCD2 in MS.

Parkinson′s Disease (PD) is the second most common neurodegenerative disease in older adults and consists of the degeneration of the dopaminergic innervation of the basal ganglia [60,61]. The major symptoms of PD include resting tremor, extrapyramidal rigidity or hypertonia, bradykinesia or akinesia, postural instability, freezing of gait, sialorrhea, amimia, depression, and cognitive impairment [62]. α-Synuclein (αS), a 14 kDa peptide, is an abundant nerve cell component that forms abnormal aggregates in PD and promotes lipid droplet formation [63]. Suppression of SCD was protective against αS yeast toxicity [64]. Similarly, inhibition of SCD blocked toxicity in αS-overexpressing rat neurons and deletion of SCD prevented αS-induced dopaminergic degeneration in *Caenorhabditis elegans* [64].

Recently, transgenic mice that expressed a PD-associated form of αS were treated with SCD inhibitor by oral administration and were characterized [65]. Inhibition of SCD protected these mice from gait defects, resting tremor, and progressive motor decline; however, they also exhibited hair loss and dry eyes [65]. Mice heterozygous for *Scd1* and expressing a PD-associated form of αS displayed less lipid-rich αS aggregates and performed better on rotarod tests [65]. As SCD inhibitors generally target all SCD isoforms and *Scd1* haploinsufficiency is effective in reducing PD phenotypes, specifically inhibiting either SCD2 or hSCD5 in the brain may also be a viable method for combating αS-induced neurotoxicity without unwanted side effects.

### 2.7. Human Relevance

Based upon the tissue expression and functional similarities between SCD2 and hSCD5, they are thought to be orthologs; however, hSCD1 is considered the ortholog of all murine isoforms. Unlike the murine SCD isoforms, which are located on the same chromosome, hSCD5 and hSCD1 are located on different chromosomes (4 and 10, respectively). Since hSCD1 and hSCD5 are located on separate chromosomes, it was hypothesized that hSCD1 and hSCD5 did not arise by gene duplication, similar to the murine isoforms, and that hSCD5 was a new distinct isoform [66]. Despite the evolutionary differences between hSCD5 and SCD2, SCD2 may prove to be a useful model for hSCD5, particularly in CNS diseases. hSCD5, like SCD2, is the predominant isoform in the brain and pancreas [15]. Furthermore, disruptions in SCD2 and hSCD5 are both associated with birth defects [66]. Pericentric inversion of *hSCD5* is associated with cleft lip, a common human birth defect affecting 1 out of every 700 births [67]; whereas, SCD2KO mice have caudal vertebrae defects.

One difference between SCD2 and hSCD5 is their expression in adipose tissue. Unlike the high expression level of SCD2 in murine adipose tissue, hSCD5 is expressed at low levels in human adipose tissue [66]. However, obesogenic diets may induce hSCD5 in adipose tissue similar to SCD2 in mice; but this has not been investigated. To our knowledge, there have been no direct investigations into the contribution of hSCD5 to metabolic diseases. Regardless, given the impact SCD2 deficiency has on metabolism, future studies into the role of hSCD5 are warranted. If hSCD5 behaves similarly to SCD2, then metabolic studies involving hSCD5 may lead to new treatments for the metabolic syndrome.

## 3. Materials and Methods

### 3.1. Animals and Diets

All animal studies were approved by and carried out following the Institutional Animal Care and Use Committee guidelines at The University of Wisconsin-Madison (protocol #A005125, approved 23 April 2018). Generation and maintenance of *Scd*2^−^/^−^ and *Scd1*^−^/^−^ mice have been described previously [24,25]. Generating sufficient numbers of mice for this study was a challenge and limitation because < 25% of SCD2KO mice survived the first 24 h after birth. All mice used in this study were in the 129/Sv genetic background and maintained on a 12-h light/dark cycle with free access to food and water. Mice were weaned at 21 days post birth and fed standard chow (Purina 5008). Unless otherwise indicated, mice were individually caged and fed a lard-based, high-fat diet for 10 weeks (HFD; 60% kcal from fat; Research Diets #D12492), beginning at 6 weeks of age. Before being euthanized by isoflurane overdose, all mice were fasted for 4 h.

### 3.2. Skeletal Staining

Skeletons were stained with alizarin red, as previously described [68]. Briefly, the skeletons were fixed in 95% ethanol overnight and then transferred to acetone overnight. The skeletons were then washed with 70% ethanol for 6 h and then transferred to 1% KOH for 2 days. Bone tissue was stained with 1% alizarin red in 1% KOH for 2 days. After staining, the stained skeletons were drained and placed in 100% glycerol.

### 3.3. Dual-Energy X-ray Absorptiometry (DEXA)

DEXA was performed with the PIXImus software version 2.10 (GE/Lunar Corp, Madison, WI, USA) to obtain BMD and BMC. Mice were anesthetized with isoflurane via an anesthesia machine (IsoFlo, Abbott Laboratories, North Chicago, IL, USA) and placed prone with limbs and tail stretched away from the body. The analysis of each scan excluded the head.

### 3.4. Generation of SCD1 and SCD2 Knockout 3T3-L1 Cells

CRISPR genomic editing technology was used to knock out *Scd1* and *Scd2*, respectively, in the 3T3-L1 cell line, as previously described [69]. Briefly, *Scd1* and *Scd2* guide RNA sequences were cloned into the pSpCAS9(BB)-2A-Puro (px459) vector, a gift from Feng Zhang (Addgene plasmid#41839; http://n2t.net/addgene:48139; RRID: Addgene_49139). The resulting plasmids were transfected into 3T3-L1 cells using Lipofectamine 2000 (Life Technologies, Carlsbad, CA, USA). After transfection, positively transfected cells were isolated by puromycin selection and seeded into a 96 well plate with only one cell per every two wells. After clonal expansion, knockouts were confirmed by Sanger sequencing and Western blot.

### 3.5. Primary Preadipocyte Isolation

Primary preadipocyte isolation was conducted as previously described [70]. Briefly, mice were euthanized at weaning, and fat pads were aseptically excised. The fat pads were minced with sharp scissors and placed in a sterile conical tube containing 0.1% collagenase type I, DMEM, 1 M HEPES, and 4% BSA. The homogenate was incubated in a shaking water bath for 1 h. After collagenase digestion, the cell suspension was filtered through a 20 μm nylon screen and centrifuged for 10 min at 300× *g*. The pelleted cells were then washed five times to remove all traces of collagenase. The cells were then resuspended in DMEM, 10% fetal bovine serum, penicillin (100 U/mL), and streptomycin (100 μg/mL).

### 3.6. Cell Culture and Treatments

3T3-L1 cells and primary preadipocytes were cultured in DMEM, 10% fetal bovine serum, penicillin (100 U/mL), and streptomycin (100 μg/mL). After reaching 100% confluence for 2 days, 3T3-L1 differentiation was initiated by the addition of methylisobutylxanthine (0.5 mM), dexamethasone (1 μM), and insulin (10 μM) [38]. Similarly, after reaching 100% confluence for 2 days, primary preadipocyte differentiation was initiated by the addition of methylisobutylxanthine (0.5 μM), dexamethasone (1 μM), indomethacin (200 μM), rosiglitazone (2 μM), and insulin (10 μM) [33]. After 48 h, the cells were maintained in DMEM containing 10% fetal bovine serum, antibiotics, and insulin. The medium was changed every two days. Where indicated, PPARγ agonists rosiglitazone (2 μM) and indomethacin (200 μM) were supplemented to the 3T3-L1 differentiation cocktail to more potently activate PPARγ and enhance preadipocyte differentiation. Lastly, where indicated, fatty acid free BSA-conjugated oleic acid (100 μM) or palmitoleic acid (100 μM) were supplemented to the differentiation cocktail and post-differentiation maintenance medium to mimic SCD activity. Fatty acids were prepared as previously described [71].

### 3.7. Oil Red O Staining

A stock solution of Oil Red O was prepared by dissolving 300 mg of Oil Red O powder in 100 mL of isopropyl alcohol and then filtered. Differentiated 3T3-L1 cells were gently washed with PBS and fixed in 10% formalin for 60 min at room temperature. After fixation, the formalin was removed and cells were washed with water. Next, the cells were incubated in 60% isopropyl alcohol for 5 min and then stained with 3 parts Oil Red O stock and 2 parts H_2_O. Lastly, the stain was removed and the cells were washed with water until the water was clear.

### 3.8. Quantitative Real-Time PCR

Total RNA was isolated using the RNeasy Lipid Tissue Mini Kit (Qiagen, Valencia, CA, USA). cDNA was synthesized from RNA using the ABI High Capacity cDNA Reverse Transcription Kit (Life Technologies/Invitrogen, Carlsbad, CA, USA). qPCR was performed on an ABI7500 instrument using the ABI Fast SYBR Green Master Mix (Life Technologies/Invitrogen, Carlsbad, CA, USA). Relative mRNA abundance was calculated as relative Ct value and normalized to 18 s by the ΔΔCt method. Primer sequences are available upon request.

### 3.9. Western Blot Analysis

Adipose tissue was homogenized in ice-cold RIPA buffer with protease inhibitors (Protease inhibitor Cocktail Set III, Calbiochem, La Jolla, CA). Protein concentration was quantified using a BCA protein assay (Thermo Fisher Scientific, Hanover Park, IL, USA). For immunoblots, 30 µg of protein sample was separated by 10% SDS-PAGE and transferred to a PVDF membrane (MilliporeSigma, Burlington, MA, USA). Membranes were blocked in 5% nonfat dry milk (*w/v*) and incubated with a primary antibody against PPARγ (Santa Cruz Biotechnology, sc-7273, Santa Cruz, CA, USA) or GAPDH (MAB374, Sigma-Aldrich, St. Louis, MO, USA), followed by IgG-horseradish peroxidase-conjugated secondary antibody. Blots were developed using Amersham ECL Prime Detection Reagent (VWR, West Chester, PA, USA).

## 4. Conclusions

SCD2 is a critical enzyme in murine development, metabolism, and disease. Here we provided a comprehensive review on SCD2 and showed that SCD2KO mice have decreased BMD and BMC when fed a HFD. Additionally, we showed that SCD2 is not required for PPARγ expression and preadipocyte differentiation in vivo, despite being required in vitro.

Since SCD2 was first identified, our knowledge about its physiological role has grown significantly; yet, many questions remain such as why does SCD2 deficiency create dramatic bone abnormalities in the tails of developing mice? Can adipose tissue-specific deletion of SCD2 alone recapitulate the beneficial metabolic effects exhibited by SCD2KO mice, or is a simultaneous deletion of SCD1 and SCD2 required to produce an observable phenotype? Why does SCD2 regulate preadipocyte differentiation via PPARγ in vitro but not in vivo? Can SCD2 knockout and overexpression mouse models serve as tools to study MS, AD, and PD treatments? Does targeting hSCD5 lead to metabolically beneficial effects similar to SCD2? Answering these questions may provide the groundwork for new drug treatments for metabolic and CNS diseases.

Drugs targeting SCD1 have shown promising effects in mice to prevent Parkinson′s disease, blunt weight gain, improve glucose tolerance, promote sebaceous gland atrophy, protect against fatty liver disease, and inhibit cancer cells [65,72]. Despite this, topical and intraperitoneally injected SCD inhibitors produce unwanted side effects, such as dry eyes, partial eye closure, dry skin, and alopecia [45,65,72,73]. These side effects do not occur in SCD2KO mice, and therefore SCD2 may be a more suitable drug target than SCD1.

## Abbreviations

AC	Acylceramide
ACC	Acetyl-CoA carboxylase
AD	Alzheimer′s disease
ATF4	Activating transcription factor 4
BAT	Brown adipose tissue
CE	Cholesterol ester
CEBP	CCAAT/Enhancer binding protein
ChREBP	Carbohydrate response element-binding protein
CKD	Chronic kidney disease
CNS	Central nervous system
DGAT2	Acyl-CoA:diacylglycerol acyltransferase-2
ER	Endoplasmic reticulum
FA	Fatty acid
FAS	Fatty acid synthase
FFA	Free fatty acid
GBA	β-cerebrosidase
hSCD1	Human stearoyl-CoA desaturase-1
hSCD5	Human stearoyl-CoA desatuarse-5
MS	Multiple sclerosis
mTOR	Mammalian target of rapamycin
MUFA	Monounsaturated fatty acid
PA	Phosphatidic acids
PD	Parkinson′s Disease
PGC1α	Peroxisome proliferator-activated receptor gamma, coactivator 1 alpha
PL	Phospholipid
PPARγ	Peroxisome proliferator-activated receptor gamma
SCD	Stearoyl-CoA desaturase
SCD1	Stearoyl-CoA desaturase-1
SCD2	Stearoyl-CoA Desaturase-2
SCD3	Stearoyl-CoA desaturase-3
SCD4	Stearoyl-CoA desaturase-4
SFA	Saturated fatty acid
SREBP1c	Sterol regulatory element-binding protein-1c
TG	Triglyceride
UCP1	Uncoupling protein-1
VC	Vascular calcification
WAT	White adipose tissue
